# Rethinking the role and impact of health information technology: informatics as an interventional discipline

**DOI:** 10.1186/s12911-016-0278-3

**Published:** 2016-03-29

**Authors:** Philip R. O. Payne, Yves Lussier, Randi E. Foraker, Peter J. Embi

**Affiliations:** College of Medicine Department of Biomedical Informatics, The Ohio State University, Columbus, OH USA; College of Medicine, Center for Biostatistics and Biomedical Informatics, University of Arizona, Tucson, AZ USA; College of Public Health, Division of Epidemiology, The Ohio State University, Columbus, OH USA

**Keywords:** Biomedical research, Informatics, Research design

## Abstract

Recent advances in the adoption and use of health information technology (HIT) have had a dramatic impact on the practice of medicine. In many environments, this has led to the ability to achieve new efficiencies and levels of safety. In others, the impact has been less positive, and is associated with both: 1) workflow and user experience dissatisfaction; and 2) perceptions of missed opportunities relative to the use of computational tools to enable data-driven and precise clinical decision making. Simultaneously, the “pipeline” through which new diagnostic tools and therapeutic agents are being developed and brought to the point-of-care or population health is challenged in terms of both cost and timeliness. Given the confluence of these trends, it can be argued that now is the time to consider new ways in which HIT can be used to deliver health and wellness interventions comparable to traditional approaches (e.g., drugs, devices, diagnostics, and behavioral modifications). Doing so could serve to fulfill the promise of what has been recently promoted as “precision medicine” in a rapid and cost-effective manner. However, it will also require the health and life sciences community to embrace new modes of using HIT, wherein the use of technology becomes a primary intervention as opposed to enabler of more conventional approaches, a model that we refer to in this commentary as “interventional informatics”. Such a paradigm requires attention to critical issues, including: 1) the nature of the relationships between HIT vendors and healthcare innovators; 2) the formation and function of multidisciplinary teams consisting of technologists, informaticians, and clinical or scientific subject matter experts; and 3) the optimal design and execution of clinical studies that focus on HIT as the intervention of interest. *Ultimately, the goal of an “interventional informatics” approach can and should be to substantially improve human health and wellness through the use of data-driven interventions at the point of care of broader population levels. Achieving a vision of “interventional informatics” will requires us to re-think how we study HIT tools in order to generate the necessary evidence-base that can support and justify their use as a primary means of improving the human condition.*

## Background

Much has been written in the contemporary scientific literature and general media concerning the promise of widespread adoption and use of Health Information Technology (HIT). The reported benefits of HIT include improved healthcare quality and safety, decreased costs, and an enhanced ability to conduct research that can, in turn, inform new approaches to healthcare delivery and wellness promotion [1, 2]. More recently, the United States government has announced a Precision Medicine initiative, which includes as part of its objectives the rapid translation of scientific knowledge generated via research programs into actionable, evidence-driven interventions aimed at improving the health of individuals and populations [3, 4]. Taken as a whole, these efforts represent an exciting inflection point in the history of the health and life sciences. It is our perspective that the power of data analytics can be harnessed in such a context to fundamentally reshape the ways in which we care for patients, and perhaps more importantly, prevent disease in the first place.

In recent commentaries, we have described the need for a healthcare system that enables not only evidence-based medicine (EBM) but also evidence-generating medicine (EGM), wherein the practice of medicine, systematic learning, and the promotion of health become cyclical, synergistic and data-centric [5]. Even as such a system relies upon robust information systems, concerns have been raised about the capabilities and functionality of current HIT platforms to meet such aspirational goals. These critiques have included concerns about: 1) the effective end-user adoption and utilization of clinical decision support systems [6–9]; 2) challenging interfaces and usability issues surrounding HIT platforms that may have a negative impact on “real world” workflow and productivity [2, 9, 10]; and 3) restrictive vendor behaviors that make it difficult to leverage HIT systems for the purposes of integrating and interacting with diverse and complex data types [11–13]. At a high level, these issues speak to a confluence of multiple important areas that impact the use and utility of HIT, including but not limited to healthcare delivery science, biomedical informatics, human factors, cognitive science, and socio-cultural engineering. While we will not be able to address each of the aforementioned areas in a comprehensive manner, we will attempt to address them in this commentary in a broad manner by discussing the types of characteristics that define the present healthcare data and information technology “ecosystem”, specifically:

1. Rapid and broadening adoption of HIT platforms provides a technological basis for improving our healthcare delivery and research enterprises;
2. Such platforms will become even more important to the delivery of care and the promotion of wellness as we seek to adopt a Precision Medicine approach;
3. The healthcare and life science community are in need of a well informed and supported evidence-base that can support and enable the use of HIT as a primary means of impacting individual patients and their communities; and
4. Creating such an evidence-base will require the study of HIT in a manner that allows for the focused evaluation of data-driven interventions as a primary end-point, while simultaneously seeking to understand the complex technology, human, and environmental issues that predispose or influence such strategies.

It is this last characteristic of the HIT ecosystem that is the our focus, namely the need for a new approach to clinical research that enables the generation of the required evidence-base that will in turn allow us to more effectively and comprehensively use HIT to impact individual patients and their communities from a health and wellness standpoint. We will refer to this type of approach as Interventional Informatics (I^2^), and define it as follows:

*Interventional Informatics (I*^*2*^*) an approach to using HIT in a manner that improves clinical decision-making, care delivery processes and/or population health strategies while simultaneously enabling systematic evidence generation through routine practice.*

Given such a working definition, *it can be seen that the primary objective of I*^*2*^*is to enable quantifiable, patient-centric improvements in health and wellness through the use of precision, data-driven interventions.* In this way, *I*^*2*^ emphasizes an active and patient-centric use of HIT that can impact health and wellness in a measurable manner, analogous to more traditional approaches such as medications or clinical procedures. This stands in contrast to traditional studies where Biomedical Informatics theories and methods are used to inform, support, or facilitate the generation of new clinical or population health evidence, but are not used as the primary intervention strategy (e.g., where technology and data support or enable a diagnostic or therapeutic activity) . In this manner, it is the proximity to patients, and the goal of directly impacting health outcomes that delineates what is and is not *I*^*2*^.

## Discussion

### Introducing interventional informatics: a new approach to studying primary hit interventions to improve human health and wellness

**Fig. 1:**
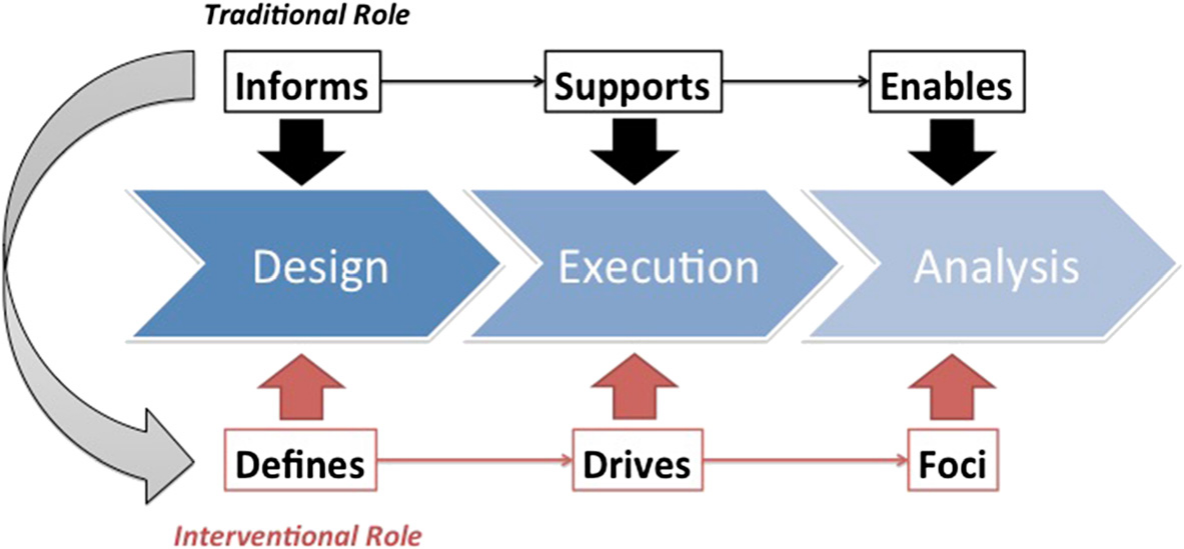
Overview of the transition required from a traditional role for informatics in healthcare research and innovation to one that is interventional in nature. The core of this model is the rethinking of the type of evidence to be generated, and the corresponding focus of studies contributing to such a knowledge base. In this figure, we compare and contrast the way in which informatics theories and methods apply to the design, execution, and analysis of a given area of inquiry, shifting from a support or enabling role to one that is the direct and primary focus of ensuing research programs

As was noted previously, in most contemporary clinical studies, the prevailing paradigm for the application of informatics theories and methods follows a pattern such that theories and methods are utilized to: 1) inform the design of a study focusing on a traditional intervention (e.g., a drug, device, or diagnostic method); 2) support the collection and management of data generated during the execution of that study; and 3) facilitate the analysis of such data and determination of findings that serve to advance the biomedical knowledge base [14]. *The defining role of informatics in this traditional paradigm is that of being a secondary substrate upon which primary interventions are delivered, studied and empirically justified*. In contrast, the I^2^ approach we have defined herein promotes the use of informatics theories and methods to: 1) design and implement the primary intervention(s) to be studied; 2) drive the execution of such intervention(s) via the delivery and instrumentation of technology-based mechanisms to promote health or manage the treatment of disease; and 3) contextualize and focus the analysis of ensuing results. These steps in aggregate are intended to establish an evidence-base via which informatics interventions become a primary means of impacting patient health and wellness, rather than serving in a facilitating role. The creation and widespread use of such an evidence base has significant benefits in scenarios amenable to technology-based interventions, wherein the time, cost, and failure rate of generating and applying effective clinical interventions can be substantially reduced, while simultaneously improving the return-on-investment and patient benefits afforded by widespread HIT adoption. A comparison of these two approaches as they relate to a high-level research project design and ensuing evidence generation is shown in Fig. 1.

### Informatics as the intervention: rethinking the Clinical Research Paradigm to develop an I^2^ evidence base

**Fig. 2:**
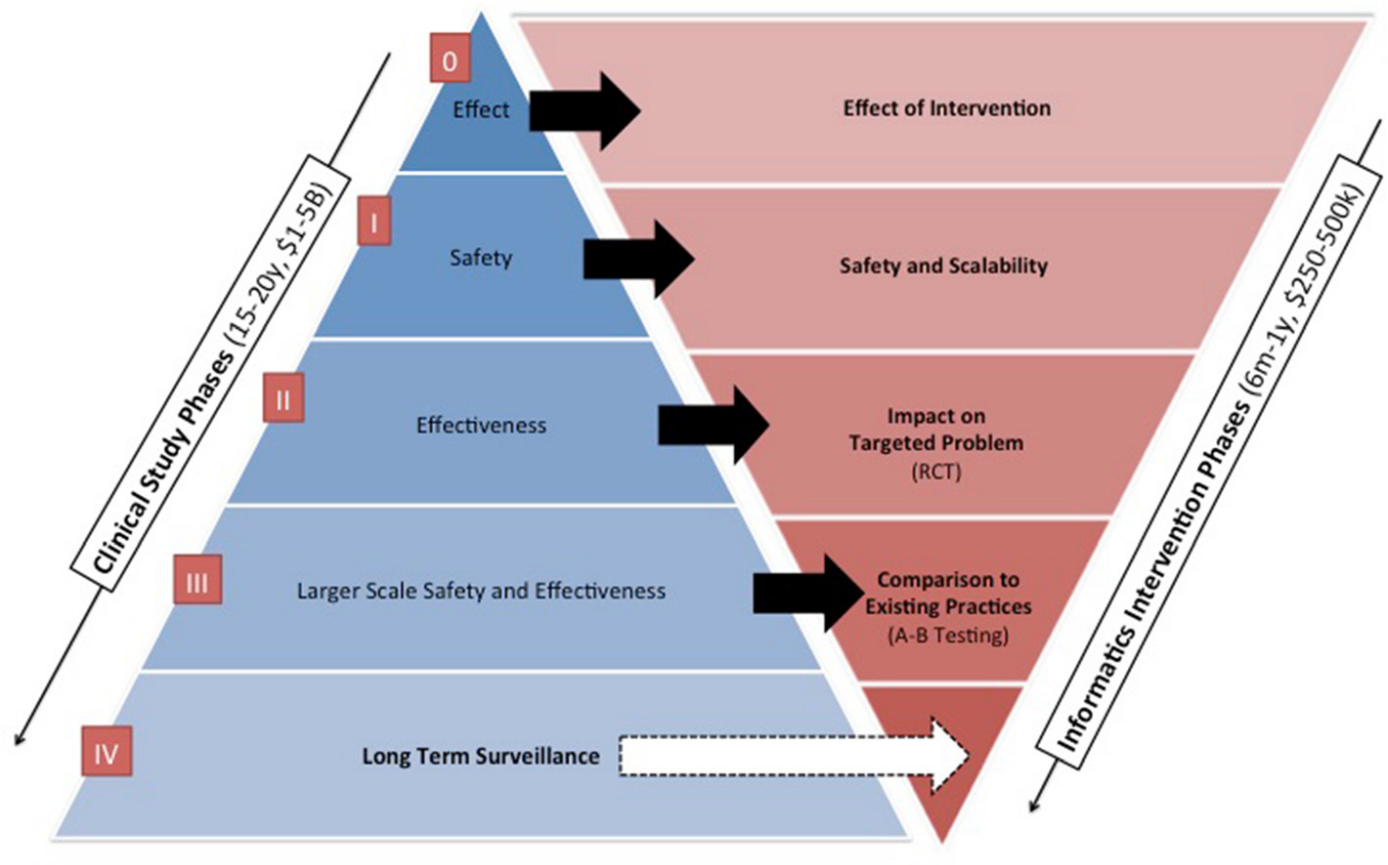
Overview of the “Interventional Informatics” paradigm, aligned with traditional clinical study phases and outcomes. These overall approaches are also contrasted relative to their average cost and timeliness. In this figure, we show how traditional types of research questions commonly pursued in a clinical trial or study can be modified and applied to enable the study of interventions that are primarily data-driven and technology focused

Implementing an I^2^ approach in order to generate the requisite evidence for the use of HIT as a primary intervention will require the extension and enhancement of traditional clinical research frameworks that focus on traditional therapeutic agents, devices, and diagnostic methods, in order to fully account for the nature of technology-based intervention strategies. A comparison and discussion of potential points of contrast between traditional and I^2^-centric approaches to clinical research is provided below, and illustrated in Fig. 2. Of note in regards to this figure is an approximate comparison of the timelines and resources associated with traditional clinical studies as contrasted with an I^2^-centric approach wherein an intervention may be thought of as a data-driven application or “app.” We believe that the magnitude of this comparison is valid, while simultaneously acknowledging that due to the limited sources and biases of the available knowledge base (e.g., having being generated via non-scholarly evaluations of the field) that serves to define such information, such data points represent a rough approximation at best.

Phase 0: In a traditional clinical study, early stage research (Phase 0 or pre-clinical phase) focuses on the effect or measurement capabilities of the new therapy or diagnostic approach relative to the physiological phenomena of interest. This stage tends to build on substantial prior basic science research. Outcomes often involve the measurement of direct or surrogate physiological processes or products. In an I^2^ paradigm, the basic premise of this phase would remain the same, with a potential refocusing on broader systems-based phenomena as an alternative to a pathophysiologic state. For example, such a systems-level approach could involve the interplay of patients, providers, and Electronic Health Records (EHRs) as they are combined to enable the tailored delivery of information and its impact on modifiable risk factors for a given disease state. Outcomes could include end-user acceptance and/or usability of the intervention as a surrogate for the ability of stakeholders to adopt and effectively use the new technology.

Phase I: In a traditional clinical study, the research questions addressed in a Phase I study usually focus on the safety of the new therapy or diagnostic approach. In an I^2^ paradigm, safety would still remain a focus, as would scalability of the technology, which could directly impact safety in later study phases. For example, if a technology-based intervention that uses a complex algorithm is computationally tractable in small scale studies (being computable in a sufficiently short time frame so as to be usable at the point-of-care), but cannot be reasonably calculated for larger numbers of patients in real-time, then substantial safety concerns would be raised in terms of delivering said intervention for use in general clinical practice. This is the type of question that can and should be addressed in Phase I of an I^2^ paradigm study.

Phase II: When conducting a traditional clinical study, Phase II research is often concerned with the effectiveness of the diagnostic or therapeutic approach being studied. Such efficacy is normally measured by using one or more markers for the presence of disease or response to therapy (e.g., bio-markers). In a similar manner, the I^2^ paradigm can be used to study efficacy of a technology-based intervention. Such efficacy can be measured via a data-driven marker, such as those associated with measurable clinical phenotype characteristics or patient reported outcomes. As a result, the goal of this phase is to identify whether the technology intervention is having an impact or effect on the desired physiological, behavioral, or other end-point targeted by the I^2^ approach.

Phase III: The I^2^ paradigm diverges more substantially from traditional Phase III research designs. In a normative clinical trial, Phase III research would extend Phase II to compare outcomes or indicators of efficacy with a gold standard, such as a prevailing treatment or diagnostic modality. In this scenario, a pair-wise comparison is used to examine two comparable approaches that address a common driving clinical problem. In contrast, the Phase III of the I^2^ approach compares the performance of a technology-based intervention with a conventional technique (such as a drug or qualitative judgment conducted by a physician). Such a comparison of the relative benefits of the informatics-based and non-informatics based approaches satisfies what is commonly known as a comparative effectiveness research question, which traditionally involves assessments of comparative benefits in areas such as clinical outcome, cost, and quality.

Phase IV: Finally, in both a traditional clinical study as well as in the I^2^ paradigm, Phase IV or “post-market” research involves the long term and longitudinal monitoring of the effects of a diagnostic or therapeutic approach as observed in an a population situated in normative settings (e.g., as part of routine care or health promotion activities), as opposed to a research context.

### An example of the I^2^ paradigm: the SPHERE project

As was noted previously, EHR platforms hold great promise relative to the ability to improve patient care and outcomes, especially when incumbent clinical decision support (CDS) functionality is used to address modifiable and measurable risk factors for disease onset or progression. A primary example of a driving clinical problem in which these types of factors are of great importance is the management of cardiovascular disease (CVD) risk. EHR-based interventions in this realm have a strong evidence base in CVD prevention and the potential to improve clinical decision-making and healthcare delivery while maintaining an efficient healthcare encounter workflow. To this end, interventional informatics technology with a flexible, robust architecture can be used to deliver precision medicine on a large scale.

However, and as was introduced previously, there are a variety of barriers to the efficient use of clinical data collected via an EHR at the point-of-care so as to support risk identification and management. For example, relevant vital signs, laboratory results, medication usage, and health behavior indicators are often captured in multiple structured, semi-structured, and unstructured formats that are distributed throughout a variety of screens and interfaces in most commonly used EHR platforms. Further, even if such data are identified and used for a CDS alerting strategy, the ways in which ensuing alerts can be programmed and delivered to the end-user are usually relatively simplistic (involving basic logical operations applied to structured data elements and the presentation of the alert using textual user interface elements). These types of challenges are representative of an even broader spectrum of potential barriers to the satisfaction of clinical and patient information needs, human-computer interaction optimization, and workflow integration, not to mention missed opportunities to engage patients in meaningful dialogues relative to clinical decision-making and health promotion.

In response to these types of challenges and opportunities, and motivated by the clinical importance of CVD risk management, we developed and studied an EHR-integrated tool whereby CVD data are integrated, analyzed, and presented using state-of-the-art visualization methods [15]. This technology, known as the Stroke Prevention in Healthcare Delivery Environments (SPHERE) tool, was explicitly designed from the outset with an interactive interface to enhance patient-provider communication around CVD relevant behavioral risk factors, thus providing a data-driven health and wellness intervention via which those risk factors would ideally be discussed and managed by patients, and their caregivers and healthcare providers at the point-of-care and beyond. In order to study the efficacy and impact of this patient-centric intervention, we conducted a practice-randomized study of the SPHERE tool in primary care settings. In this study design, a traditional case and control group comparison approach was employed, and the tool was assigned to comparable clinical practice settings (where the unit of randomization was the practice itself). At the conclusion of the study, we were able to identify significant and positive changes in both Body Mass Index (BMI) and Hemoglobin A1C measurements for the population of patients in the clinic that received the SPHERE intervention when compared to the control clinic. Both of these measures related to important and modifiable risk factors for CVD onset, and as such, are viable surrogates for showing the clinical efficacy of the intervention strategy. Full details concerning this study, its design, and results can be found in a number of publications [16, 17]. Furthermore, at a high level, this study is a primary example of the previously introduced I^2^ paradigm, whereby:

1. The design and implementation of *the primary intervention underlying our study was data-centric in nature* and focused upon an informatics based approach to improving population health, in this case as it relates to CVD risk;
2. The execution of our intervention focused on the delivery and instrumentation of a technology-based mechanism that leveraged extant HIT platforms, and further, *our informatics-based approach to managing disease-specific risk factors was intended to have a direct and measurable impact on a driving clinical problem*;
3. *The focus of our analysis of the study was on the efficacy and impact of said technology-based mechanism, and employed a well-validated clinical research model to generate high quality clinical evidence.*

### Challenges and opportunities surrounding interventional informatics (I^2^) research and innovation

As was introduced above, substantial opportunity exists to advance the speed and cost-effectiveness of evidence generation in support of healthcare delivery via the rigorous study of interventions that are primarily technology based. However, there remain challenges to realizing such a vision, including:

- One of the primary platforms for designing and implementing technology interventions for the clinical environment is the implementation of novel functionality within EHR systems. However, due to technical constraints, variable data and programmatic standards compliance, and restrictive licensing and usage policies, the ability to implement such interventions is both complicated and yields inconsistent generalizable success across environments and platforms. This challenge of vendor propriety, and the need to introduce technical and regulatory frameworks that overcome said barriers, have been explained in detail in recent publications [8, 11, 12]. Such measures can and should be one of the top priorities for the healthcare research and delivery communities if we are going to realize the full value of our collective technology investments to-date.
- The design and conduct of I^2^ paradigm studies will necessitate the participation of multi-disciplinary teams of investigators and clinicians, working together, and motivated by common benefits and incentive structures. However, despite over a decade of community dialogue concerning the importance of supporting and enabling team science, major cultural, workflow, resource and policy barriers impede such team formation and operation. As such, realizing the full benefits of the I^2^ paradigm will require a redoubling of efforts to address such gaps and create new modes of team formation, support and incentive structures. This will also by necessity require a full consideration of critical environmental, funding, and policy issues that impact such collaborative efforts. A glimpse of how to address these types of challenges is provided in a recent publication by one of the authors (PJE) concerned with the need for a financial model that encourages and enables participation in research by front-line clinicians [18].
- Finally, while we have presented an overarching model for the types of inquiries to be asked and answered during the different stages of an archetypal I^2^ research program, there remains much work to be done to more fully formalize study designs and best practices that can express the range of hypotheses and research questions incumbent to this emergent model. Examples of issues here include the identification of metrics and end-points to be measured, mechanisms of managing bias (both implicit and explicit), and satisfaction of sampling and reproducibility concerns through appropriate modeling and qualification of results [14].

The work required for the preceding mitigating approaches can and should be motivated by the fundamental differentiation of potential costs and benefits associated with traditional clinical studies that may take upwards of 20 years and cost up to 5B USD, when compared to I^2^ paradigm studies, that can be completed in as little as 6 months with a cost around 250,000 USD based upon current survey data from the Kinvey group [10]. Furthermore, we believe that the rapidly maturing fields of study surrounding the component approaches that make up an I^2^ paradigm are well positioned to address many if not all of the preceding challenges in a timely and compelling manner, given sufficient support and attention by the research and clinical practice communities in equal measure.

## Conclusions

Advances in the adoption and use of HIT have and continue to make a dramatic impact on the practice of medicine. Building upon this, and in an effort to achieve an EGM paradigm [5], it can be argued that now is the time to pursue new means of studying HIT as the interventional strategy that is needed in order to realize the promise of precision medicine. Achieving this goal will ultimately require the health and life sciences communities to embrace a new mode of using and studying HIT, which we have introduced here as “interventional informatics” or I^2^. Achieving this vision will require our community to address critical issues, including the: 1) nature of the relationships between HIT vendors and healthcare innovators; 2) formation and function of multidisciplinary teams consisting of technologists and clinical or scientific subject matter experts; and 3) optimal design and execution of clinical studies that focus on HIT as the intervention of interest, including a thorough understanding of the resource and financial needs underlying such investigations. Furthermore, addressing these types of open questions will also require thoughtful consideration of how the “interventional informatics” or I^2^ paradigm is best positioned in the taxonomic definitions that serve to organize the broader field of Biomedical Informatics, and in particular, whether such an approach to research constitutes a new sub-discipline of the field, an extension to an existing sub-discipline such as Clinical Research Informatics (CRI), or a cross-cutting experimental framework. While a full treatment of all of the preceding challenges and opportunities is beyond the scope of a single commentary such as this, it is our hope that by raising the fundamental questions required to implement and pursue studies aligned with an I^2^ paradigm, we can catalyze the necessary community dialogue and ensuing efforts to overcome such barriers. Ultimately, while there exist substantial challenges to achieving an I^2^ paradigm, a concerted and focused effort to address these issues has the potential to yield substantive and highly impactful changes in the healthcare system by which technology assumes a primary role in delivering high quality, safe, and cost effective clinical care and wellness promotion, and is thus worthy of further pursuit, energy, and effort.

### Ethics approval and consent to participate

Not applicable.

### Consent for publication

Not applicable.

### Availability of data and materials

This publication represents the viewpoints of the authors, and does not involve the direct generation of any data or materials.

## Abbreviations

EBM: Evidence-based medicine
EGM: Evidence-generating medicine
EHR: Electronic health record
HIT: Healthcare information technology
I2: Interventional informatics
USD: United States Dollar
